# Agricultural mulching and fungicides—impacts on fungal biomass, mycotoxin occurrence, and soil organic matter decomposition

**DOI:** 10.1007/s11356-021-13280-3

**Published:** 2021-03-11

**Authors:** Maximilian Meyer, Dörte Diehl, Gabriele Ellen Schaumann, Katherine Muñoz

**Affiliations:** iES Landau, Institute for Environmental Sciences Landau, Group of Environmental and Soil Chemistry, University Koblenz-Landau, Landau, Germany

**Keywords:** Plastic mulching, Fenhexamid, Cyprodinil, Fludioxonil, Deoxynivalenol

## Abstract

Plastic and straw coverage (PC and SC) are often combined with fungicide application but their influence on fungicide entry into soil and the resulting consequences for soil quality are still unknown. The objective of this study was to investigate the impact of PC and SC, combined with fungicide application, on soil residual concentrations of fungicides (fenhexamid, cyprodinil, and fludioxonil), soil fungal biomass, mycotoxin occurrence, and soil organic matter (SOM) decomposition, depending on soil depth (0–10, 10–30, 30–60 cm) and time (1 month prior to fungicide application and respectively 1 week, 5 weeks, and 4 months afterwards). Soil analyses comprised fungicides, fusarium mycotoxins (deoxynivalenol, 15-acetyldeoxynivalenol, nivalenol, and zearalenone), ergosterol, soil microbial carbon and nitrogen, soil organic carbon, dissolved organic carbon, and pH. Fludioxonil and cyprodinil concentrations were higher under SC than under PC 1 week and 5 weeks after fungicide application (up to three times in the topsoil) but no differences were observed anymore after 4 months. Fenhexamid was not detected, presumably because of its fast dissipation in soil. The higher fludioxonil and cyprodinil concentrations under SC strongly reduced the fungal biomass and shifted microbial community towards larger bacterial fraction in the topsoil and enhanced the abundance and concentration of deoxynivalenol and 15-acetyldeoxynivalenol 5 weeks after fungicide application. Independent from the different fungicide concentrations, the decomposition of SOM was temporarily reduced after fungicide application under both coverage types. However, although PC and SC caused different concentrations of fungicide residues in soil, their impact on the investigated soil parameters was minor and transient (< 4 months) and hence not critical for soil quality.

## Introduction

Mulching techniques such as straw and plastic mulching have become important agricultural practices to improve crop growth conditions in order to increase agronomic productivity (Haapala et al. 2014; Iqbal et al. 2020). Strawberry cultivation typically uses mulching techniques, which improve growth conditions by increasing soil temperature and reducing evaporation, weed growth, and erosion (Iqbal et al. 2020; Steinmetz et al. 2016). The conventional straw mulching is still applied today in matted row systems, particularly in colder regions, because of its low costs and labor intensity (Daugaard 2008; Lille et al. 2003; Poling 2016; Zhou et al. 2016). Because plastic mulching mostly performs better on the aforementioned attributes than straw mulching (e.g., Gao et al. 2019; Li et al. 2018a, b; Qin et al. 2015; Yang et al. 2018), it has become often combined with ridge-furrow systems and subsurface drip irrigation, a widely applied agricultural practice in strawberry cultivation, which has largely replaced straw mulching (Kasirajan and Ngouajio 2012; Zhou et al. 2016). However, plastic mulching can also increase plastic residues and pesticide runoff, reduce SOM, and shift the microbial community towards mycotoxigenic fungi (reviewed in Steinmetz et al. 2016), which has recently raised concerns about the sustainability of the system (Steinmetz et al. 2016). In particular, two aspects are still missing about plastic mulching, which are necessary to evaluate its impact on soil quality in the long term: (1) its potential to contaminate soil with microplastics and mycotoxins and (2) a substantial process understanding of its influence on various soil parameters and processes (Accinelli et al. 2020; Steinmetz et al. 2016).

Fungicides are widely applied in agriculture to protect crops against fungal diseases and are frequently combined with plastic and straw mulching. They can reach the soil either by direct application or indirectly by drift during spraying or runoff from sprayed plants after precipitation or irrigation (Arias et al. 2005; Cisar and Snyder 1996; Wainwright 1977). Fungicides are known to impact directly or indirectly on a multitude of soil processes. For example, fungicides reduce fungal population by inhibiting growth and sporulation (Campos et al. 2014). This can result in a reduced microbial activity (Chen et al. 2001a; Chen and Edwards 2001), a shifted microbial community (Munier-Lamy and Borde 2000; Sigler and Turco 2002; Yang et al. 2011), and a reduced genetic diversity of soil bacteria and fungi, leading to restricted enzyme activities in soil (Baćmaga et al. 2016, 2020; Monkiedje 2002; Tu 1993). Thus, relevant soil biogeochemical process can be indirectly affected by the use of fungicides, such as soil organic matter (SOM) decomposition (Chen et al. 2001a; Chen and Edwards 2001; Zaller et al. 2016) or nitrogen mineralization and nitrification (Chen et al. 2001b; Domsch 1964; Monkiedje 2002). Furthermore, filamentous fungi are an integral part of soil microbial communities and well-known producers of secondary metabolites with biological and toxic activities such as mycotoxins (Murphy et al. 2006). Mycotoxins are biosynthesized by several fungal species of the genera *Aspergillus* spp., *Penicillium* spp., and *Fusarium* spp. (Abbas et al. 2009; Murphy et al. 2006), frequently as response to stress conditions (Ponts 2015; Schmidt-Heydt et al. 2008), which can be generated by the action of certain fungicides (Chen et al. 2019; Li et al. 2018a, b). The aforementioned effects of fungicides on soil quality depend on the residual concentration of fungicides in soil, which in turn depend on application rate and frequency as well as on physicochemical and microbial soil properties (Chen and Edwards 2001; Martınez-Toledo et al. 1998; Wainwright 1977). In addition, the soil entry of fungicides and soil residual concentrations are influenced by mulching materials, which can act, depending on the mulching material, as semipermeable or impermeable physical barrier or as a sorbent material (Guo et al. 2020; Nerín et al. 1996).

Fenhexamid, cyprodinil, and fludioxonil are commonly used fungicides in strawberry cultivation to prevent infestation with *Botrytis cinerea* (gray mold) and related fungi like *Monilinia* and *Sclerotinia* (Rosslenbroich and Stuebler 2000; Strand 2008). Their half-life dissipation time (DT_50_) was in soil under aerobic laboratory conditions < 1, 53, and 239 days for fenhexamid, cyprodinil, and fludioxonil, respectively (Agriculture, and Environment Research Unit, University of Hertfordshire 2019). However, dissipation in soil depends on factors such as soil temperature, moisture, and microbial activity (Borzì et al. 2007; Dec et al. 1997; Roberts et al. 1998), which in turn can be strongly influenced by mulching treatments (Haapala et al. 2014; Li et al. 2020; Steinmetz et al. 2016).

It can be assumed that the impermeable plastic mulch acts as physical barrier for fungicides, which impedes the entries of fungicides into soil, leading to lower residual concentrations when compared to the traditional straw mulching. Because of that, we expected different effects of both mulching types on microbial (fungal) biomass, mycotoxin occurrence, and SOM decomposition, which are important factors, influencing soil quality and fertility and hence in the long-term also productivity and sustainability of the agricultural management (Bünemann et al. 2018; Frąc et al. 2018; Kibblewhite et al. 2008).

However, this has not yet been investigated but is important to understand how mulching can impact on fungicide fate and soil quality. Derived from the proceeding information, we hypothesized the following: (i) the impermeable plastic mulch mitigates fungicide entry into soil and reduces residual concentrations of fungicides in soil compared to straw-covered soil; (ii) higher fungicide concentrations in soil will strongly reduce fungal biomass and induce higher stress level to fungi, triggering a higher mycotoxin production; (iii) the fungicide residues decelerate SOM decomposition by inhibiting and reducing soil microbial biomass. In order to close the mentioned research gaps, the objective of this study was to investigate the residual concentrations of the fungicides fenhexamid, cyprodinil, and fludioxonil in soil under plastic and straw coverage in strawberry cultivation in dependence of time (4 months) and soil depth (three soil layers) and to estimate their impact on microbial (fungal) biomass, SOM decomposition, and mycotoxin occurrence.

## Material and methods

### Site description and soil management

This study was conducted in the frame of a triennial field experiment on the influence of plastic mulching on biogeochemical soil properties and processes in strawberry cultivation. The sampling site was a commercial strawberry field in southwestern Germany (49° 11′ N, 8° 10′ E, 130 m a.s.l.), which has a temperate, humid climate with an annual average precipitation of 643 mm a^−1^ (weather station of Landau-Wollmesheim, Agrarmeteorologie Rheinland-Pfalz). According to FAO classification, the soil type was a silt loam (Anthrosol) with a texture of 7 ± 2% sand, 83 ± 5% silt, and 10 ± 3% clay (IUSS Working Group WRB 2015) and an average cation exchange capacity of 1035 ± 50 mmol kg^−1^ in the 0–60-cm soil layer. Strawberries (*Fragaria* × *ananassa*, ‘Malwina’) were transplanted in July 2016 (8 plants per m^2^), after tillage, fertilization, and establishment of a ridge-furrow system with subsurface drip irrigation and plastic-mulched ridges (black polyethylene, 50 μm) and bare furrows. The furrows were covered with wheat straw in April 2017. Fungicide application was conducted by foliar application with a tractor-mounted field sprayer and application periods were from late-May 2017 until early-June 2017, depending on bloom of the strawberries. First, the fungicide Switch® (37.5% cyprodinil and 25% fludioxonil, application ratio 1.5) was applied together with the acaricide Masai® (tebufenpyrad) with an application rate of respectively 1 and 0.375 kg ha^−1^. Two weeks later, the fungicide Teldor® (50% fenhexamid) was applied with an application rate of 2 kg ha^−1^. The relevant physicochemical properties of the applied fungicides are summarized in Table 1.

**Table 1:** Physicochemical properties of the fungicides fenhexamid, fludioxonil, and cyprodinil

Fungicide	Water solubility^1^ (mg L^−1^)	GUS leaching potential index (leachability)^1^	Vapor pressure (volatility)^1^ (mPa)	Log *K*_ow_^1^	Photolysis^1^, DT_50_ (days)	Hydrolysis^1^, DT_50_ (days)	Soil degradation, DT_50_ (field) (days)
Fenhexamid	24	− 0.42 (low)	0.0004 (low)	3.51	0.05	Stable	~ 1^2,3^
Fludioxonil	1.8	− 1.47 (low)	0.0004 (low)	4.12	10	Stable	6–21^1,4,5,6^
Cyprodinil	13	1.06 (low)	0.51 (low)	4.00	7.5	Stable	2–45^1,4,5,6^

^1^Agriculture, and Environment Research Unit, University of Hertfordshire (2019)

^2^Abbate et al. (2007)

^3^Borzì et al. (2007)

^4^Liu et al. (2011a)

^5^Liu et al. (2011b)

^6^Zhang et al. (2015)

### Experimental design and sample collection

A semicontrolled field experiment was designed that reflected current agricultural practice while enabling us to study soil processes in a homogeneous soil type and avoiding masking of treatment effects by landscape variation, edge effects, and variability in agricultural treatment (e.g., in terms of active compounds and application rates). The test field included two experimental areas (21 × 10 m). The plastic mulch was immediately removed in one area after strawberry transplantation (July 2016) and the bare soil was later covered with wheat straw (April 2017), whereas the second area was left plastic-covered. Henceforth, we refer to them as straw-covered (SC) and plastic-covered (PC) areas. Both experimental areas were treated identically regarding fertilization, irrigation, fungicide application, and strawberry transplantation.

Soil samples were taken at four dates: 1 month before fungicide treatments in late-April (25.4) and respectively 1 week, 5 weeks, and 4 months after end of the fungicide treatments in mid-June (19.6), mid-July (18.7), and mid-October (9.10). In both experimental areas, a composite sample from the ridge (five single soil cores) was collected from a subplot (1 × 1 m) in each of five randomly chosen plots (10 × 1.5 m): PC (*n* = 5) and SC (*n* = 5). Soil samples were taken equidistantly between two plants (20 cm distance to plant) from topsoil layer (0–10 cm), the root layer, representing the main root zone of the strawberries (10–30 cm), and the subsoil layer below the root zone of the strawberries (30–60 cm). In the topsoil layer, soil samples were taken with stainless steel sampling rings (*d* = 5 cm, *h* = 5 cm), whereas a boring rod (Pürckhauer) was used for both deeper layers. Soil samples were homogenized and stored at 4 °C for further analyses.

### General soil parameters

Respectively one sensor of a field measuring station (ecoTech®, Bonn, Germany) was installed at three soil depths (5, 15, and 35 cm) in both treatments, which recorded hourly soil temperature and moisture. The soil depths were chosen in accordance with the soil layers selected for soil analyses. Air temperature and precipitation data were taken from the weather station Landau-Wollmesheim (Agrarmeteorologie Rheinland-Pfalz). Unless stated otherwise, air-dried and sieved (< 2 mm) soil samples were used for subsequent analyses. Soil pH was measured in 0.01 M CaCl_2_ solution, in accordance with DIN EN 15933:2012-11. For total nitrogen (TN) determination, soil samples were oven-dried (105 °C), milled (Planetary micro mill PULVERISETTE 7 premium line, Fritsch GmbH, Idar-Oberstein, Germany), and finally analyzed with a CHNS Analyzer (vario MicroCUBE, Elementar Analysensysteme GmbH, Langenselbold, Germany).

### Determination of residual concentration of fungicides in soil

Fungicides in soil samples were quantified with liquid chromatography–high-resolution mass spectrometry (LC-HRMS, Thermo Fisher Scientific, Waltham, USA) after solid-liquid extraction. More precisely, 5 g of an air-dried, milled soil sample (Planetary micro mill PULVERISETTE 7 premium line, Fritsch GmbH, Idar-Oberstein, Germany) was weighed in in 50-mL centrifuge tubes, mixed with 15 mL methanol (LC-MS grade), and shaken for 30 min on a horizontal shaker (Kreisschüttler 3015, GFL, Burgwedel, Germany). After 10 min of ultrasonic treatment (DT 514H, Bandelin electronics GmbH & Co.KG, Berlin, Germany), the suspension was centrifuged at 3170*g* for 5 min (Universal 320, Hettich Lab Technology, Tuttlingen, Germany). Subsequently, 2 mL of the supernatant was filtered with a 0.2-μm PTFE syringe filter. Finally, 0.5 mL of the filtered extract was diluted with 0.5 mL deionized water and stored at − 20 °C until LC-HRMS measurement. Chromatographic separation was performed at room temperature in a Hypersil GOLD™ C_18_ column (100 × 2.1 mm, 1.9 μm particle size, Thermo Fisher Scientific, Waltham, USA). As mobile phase, a gradient of solvent A (water + 0.1% formic acid and 4 mM ammonium formiate) and solvent B (methanol + 0.1% formic acid and 4 mM ammonium formiate) with a flow rate of 0.2 mL min^−1^ was used: 0–1 min 10% B; 1–3 min 10 to 100% B; 3–7 min 100% B; 7–7.5 min 100 to 10% B; and 7.5–10 min 10% B. The fungicides were quantified in the positive ion mode, using the following mass-to-charge ratios: 266.0730, 266.1339, and 302.0709 for fludioxonil, cyprodinil, and fenhexamid, respectively. Fungicide concentration in soil was quantified using a matrix-matched calibration curve (0.1, 0.5, 1.0, 2.5, 5.0, 7.5, 10, 25, and 50 μg L^−1^), which consisted of a 1:1 (v/v) mixture of a methanol soil extract (extraction followed the same procedure as samples) and deionized water. Samples were considered positive when concentrations were above the lowest calibration level (LCL) of 0.1 μg L^−1^, which corresponds to a soil concentration of 0.6 μg kg^−1^. For preparation of the calibration curve, fungicide standards, purchased by Sigma-Aldrich (Taufkirchen, Germany), were weighted quantitatively and reconstituted in methanol. Furthermore, a mixture of all three fungicides in methanol (conc. 1 mg L^−1^ each) was prepared and used for spiking and calibration purposes. The method was evaluated in terms of reproducibility at two concentrations of 4 and 40 μg kg^−1^ (*n* = 5 for each concentration). Recovery values ranged between 77.7 and 91.5%, with a relative standard deviation (RSD) < 20%.

### Analysis of microbial soil parameters

Soil samples were analyzed for several microbial parameters (microbial biomass carbon (MBC) and nitrogen (MBN), MBC:MBN ratio, and ergosterol as proxy for fungal biomass) and mycotoxin occurrence to describe the impact of fungicide residues on microbial and fungal biomass and microbial community. The focus was set on fusarium mycotoxins, because *Fusarium* spp. are relevant field fungi, occurring ubiquitous in soils under the present climate conditions (Elmholt 2008; Jouany 2007). The MBC and MBN were determined with chloroform-fumigation method, which estimates the difference in C and N between fumigated and non-fumigated soil samples (Blume et al. 2016; Vance et al. 1987). In brief, field-fresh soil samples were extracted with 0.5 M K_2_SO_4_ (1:4, w/v). Twenty grams of each soil sample was extracted directly (non-fumigated) and another 20 g after 24 h of chloroform fumigation (fumigated). The filtered extracts were analyzed for carbon content with a TOC analyzer (multiNC 2011S, Analytik Jena AG, Jena, Germany) and for ninhydrin-reactive nitrogen with a UV/VIS spectrometer (Specord50, Analytik Jena GmbH, Jena, Germany) after bounding ninhydrin-reactive nitrogen with ninhydrin as described in Joergensen and Brookes (1990).

The ergosterol determination was based on the method of Gong et al. (2001). Briefly, 4 g of air-dried, milled soil (Planetary micro mill PULVERISETTE 7 premium line, Fritsch GmbH, Idar-Oberstein, Germany) was extracted with 12 mL methanol for 60 min on a horizontal shaker (Kreisschüttler 3015, GFL, Burgwedel, Germany). The suspension was sonicated for 10 min (DT 514H, Bandelin electronics GmbH & Co.KG, Berlin, Germany), subsequently centrifuged for 10 min at 2000*g* (Universal 320, Hettich Lab Technology, Tuttlingen, Germany), and finally ultracentrifuged for 3 min at 7270*g* (Micro centaur, MSE Ltd, London, UK). For ergosterol measurement, 20 μL of the supernatant was injected in a high-performance liquid chromatography system with a UV detector (HPLC-UV, HPLC 1200 series, Agilent technologies, Santa Clara, USA), which was equipped with a C_18_ LiChrospher® column (LiChrospher RP-18e, 5 μm, 100 Å, 250 × 4.6 mm, Merck KGaA, Darmstadt, Germany) and quantified ergosterol at a wavelength of 282 nm. The applied method had a limit of detection (LOD) of 0.06 mg kg^−1^.

Soil samples were analyzed for the fusarium mycotoxins deoxynivalenol (DON), 15-acetyldeoxynivalenol (15-ADON), nivalenol (NIV), and zearalenone (ZEN). Principally, mycotoxin analysis was based on Mortensen et al. (2003) with small modification (Muñoz et al. 2017). In brief, 5 g of air-dried, milled soil (Planetary micro mill PULVERISETTE 7 premium line, Fritsch GmbH, Idar-Oberstein, Germany) was extracted with 15 mL methanol:water mixture (9:1, v/v) for 30 min on a horizontal shaker (Kreisschüttler 3015, GFL, Burgwedel, Germany) and subsequently for 10 min with ultrasonication (DT 514H, Bandelin electronics GmbH & Co.KG, Berlin, Germany). The suspension was centrifuged for 10 min at 2000*g* (Universal 320, Hettich Lab Technology, Tuttlingen, Germany). An aliquot of 10 mL was subsequently evaporated to dryness under a nitrogen stream at 50 °C (Evaporatorsystem EVA-EC1-24-S, VLM Korrosions-Prüftechnik, Labortechnik & Dienstleistungen GmbH, Bielefeld, Germany). One milliliter of mobile phase (methanol:water 1:1 v/v with 0.1% formic acid and 4 mM ammonia formiate) was used to reconstitute the residues. This solution was ultracentrifuged for 5 min at 7270*g* (Micro centaur, MSE Ltd, London, UK) and subsequently 20 μL of the supernatant was injected the LC-HRMS (Thermo Fisher Scientific, Waltham, USA), using the aforementioned Hypersil GOLD™ column for mycotoxin analysis. The mycotoxins were quantified with a matrix-matched calibration curve (1, 2.5, 5, 10, 25, 50, 75, and 100 μg L^−1^), which was prepared in soil extract (extraction followed the same procedure as samples). All calibration standards for mycotoxins were purchased by Romer Labs Deutschland GmbH (Butzbach, Germany). Samples were considered positive when concentrations were above the LCL, which was 2.5 μg L^−1^ for 15-ADON and 1 μg L^−1^ for DON, NIV, and ZEN, respectively, corresponding to a soil concentration of 0.75 and 0.3 μg kg^−1^. For DON and NIV, ^13^C-labeled internal standards were used as additional confirmation step. All mycotoxins were quantified in the negative ion mode (exception 15-ADON), using the following mass-to-charge ratios: 356.1750 and 272.1701 for ^13^C-DON and ^13^C-NIV and 341.1240, 357.1195, 339.1430, and 317.1389 for the DON, NIV, 15-ADON, and ZEN, respectively.

### Characterization of soil organic matter

In order to estimate the impact of fungicide residues on SOM and SOM decomposition, we examined soil organic carbon (SOC), dissolved organic carbon (DOC), MBC:SOC ratio, and C:N ratio. The SOC was measured in air-dried, milled soil samples with CHNS Analyzer (vario MicroCUBE, Elementar Analysensysteme GmbH, Langenselbold, Germany). An averaged carbonate content, determined by acid fumigation in accordance with Harris et al. (2001), was used to obtain SOC values from total C content measured by CHNS analyses. The DOC was determined in filtrated soil extracts (0.45 μm, 1:5 soil-to-water ratio, w/v) of field-fresh soil samples with a TOC analyzer (multiNC 2011S, Analytik Jena AG, Jena, Germany) in accordance with DIN EN 1484:1997-05. The C:N ratio corresponds to the SOC divided by the TN of the same sample.

### Data analyses

Correlation between two variables was calculated with Pearson’s correlation coefficient or Spearman’s rho if normality distribution was not given. For correlation analysis, only positive samples were used, with regard to fungicide and mycotoxin detection. In order to determine significant differences between means, a mixed factorial ANOVA design was used, with time and soil depth as repeated factors and treatment as fixed factor. If significant interaction effects occurred, an additional ANOVA was applied to locate significant differences, with least significance distance (LSD) testing as post hoc test. Normality distribution of data and of residuals was examined graphically with histograms and quantile-quantile plots. Variance homogeneity was checked by Levene’s test. If the probability of error was < 0.05, the differences were termed as statistically significant. Method validation for ergosterol, mycotoxin, and fungicide determination, with regard to repeatability, recovery, linearity, and range, and LOD calculation of ergosterol were based on ICH guideline Q2. The LOD was calculated as 3.3*σ*/*S*, with *σ* as the standard deviation of the intercept of the regression line and *S* as the slope of the regression line calculated from the calibration standards (International Council for Harmonisation of Technical Requirements for Pharmaceuticals for Human Use 2005). For fungicide and mycotoxin determination, the LCL of the matrix-matched calibration curve which gives a clear identifiable peak was used as LOQ. This method is based on visual (empirical) evaluation, which has been suggested to provide more realistic values in complex matrices (Şengül 2016). For all results below the LCL, the LCL/2 was used for mean calculation (Ogden 2010). IBM SPSS Statistics 25 and Microsoft Excel 2010 were used for all statistical analysis.

## Results

### Residual concentrations of fungicides in soil under plastic and straw coverage

**Fig. 1:**
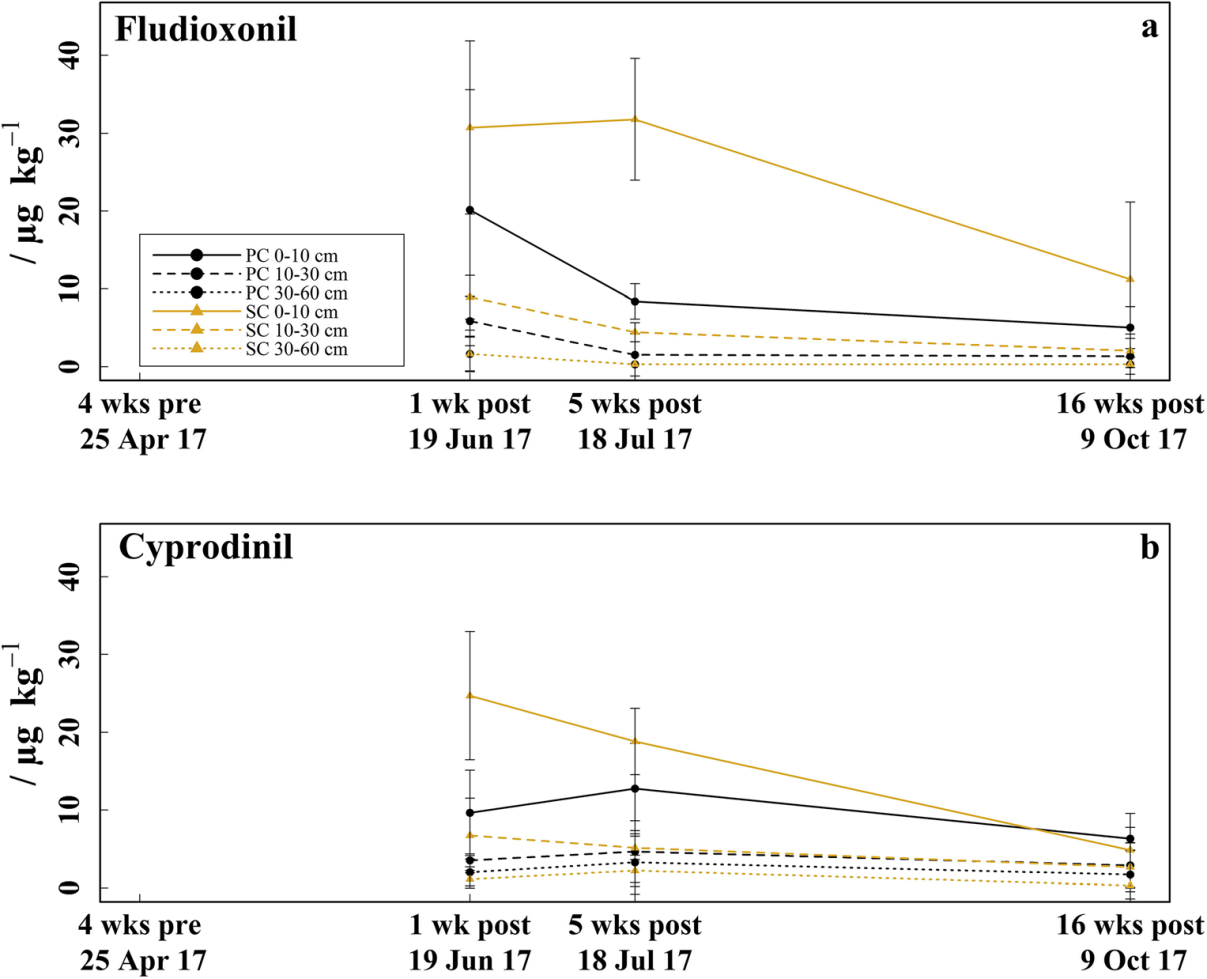
Fungicide residual concentrations in soil. **a** Fludioxonil concentrations determined 4 weeks before (late-April) and respectively 1 week (mid-June), 5 weeks (mid-July), and 16 weeks (mid-October) after fungicide application in the 0–10, 10–30, and 30–60 cm soil layer under plastic coverage (PC) and straw coverage (SC), respectively, shown as mean with standard deviation (*n* = 5). **b** Cyprodinil concentrations

The fungicide soil concentrations measured 1 week, 5 weeks, and 4 months after fungicide application (mid-June, mid-July, and mid-October) were between LCL–31.8 μg kg^−1^ for fludioxonil (Fig. 1a) and LCL–24.7 μg kg^−1^ for cyprodinil (Fig. 1b). One month (late-April) before fungicide application, neither fludioxonil nor cyprodinil was detected in any soil sample. No fenhexamid was found in any soil sample.

The fludioxonil and cyprodinil concentrations were up to three times higher under SC than under PC in the 0–10 and 10–30 cm soil layer 1 week (significant for cyprodinil in the 0–10 cm soil layer: *p* = 0.011) and 5 weeks after fungicide application (significant for fludioxonil in the 10–30 cm soil layer: *p* = 0.002). However, no differences were observed anymore between treatments 4 months after fungicide application (only the fludioxonil concentrations in the 0–10 cm soil layer under SC were still twice as high as under PC). Under SC, the fludioxonil and cyprodinil concentrations decreased by 64–82% and by 61–80%, respectively, in all soil layers from mid-June to mid-October (significant in the 0–10 cm soil layer for fludioxonil and cyprodinil: *p* < 0.030). Under PC, fludioxonil declined also by 75–82% in all soil layers from mid-June to mid-October but the cyprodinil concentrations increased by 32–64% in all soil layers from mid-June to mid-July and decreased by 38–86% to mid-October. The decline of fludioxonil was 2–23 times stronger in all soil layers under both treatments between mid-June and mid-July than between mid-July and mid-October (exception: 0–10 cm soil layer under SC). Both the fludioxonil and the cyprodinil concentrations decreased significantly with soil depth in both treatments (*p* < 0.018 and *p* < 0.040, respectively).

Despite applied in lower amounts, fludioxonil showed a tendency to higher soil concentrations compared with cyprodinil, especially under SC. In mid-June, the cyprodinil:fludioxonil ratios of both treatments ranged between 0.5 and 1.2 and were thus below the applied ratio of 1.5 (Table 2). Under SC, the cyprodinil:fludioxonil ratios remained below 1.5 in all soil layers in mid-July and mid-October (exception: 30–60 cm soil layer in mid-July), whereas under PC, the cyprodinil:fludioxonil ratios ranged between 1.5 and 10.9 above the applied ratio (exception: 0–10 cm soil layer in mid-October).

**Table 2:** Cyprodinil:fludioxonil ratios measured 1 week (mid-June), 5 weeks (mid-July), and 16 weeks (mid-October) after fungicide application in the 0–10, 10–30, and 30–60 cm soil layer under plastic coverage (PC) and straw coverage (SC), respectively

	PC			SC		
Date	0–10 cm	10–30 cm	30–60 cm	0–10 cm	10–30 cm	30–60 cm
Mid-June	0.5	0.6	1.2	0.8	0.8	0.7
Mid-July	1.5	3.1	10.9	0.6	1.2	7.3
Mid-October	1.3	2.2	5.6	0.4	1.3	1.0

### General soil parameters

**Fig. 2:**
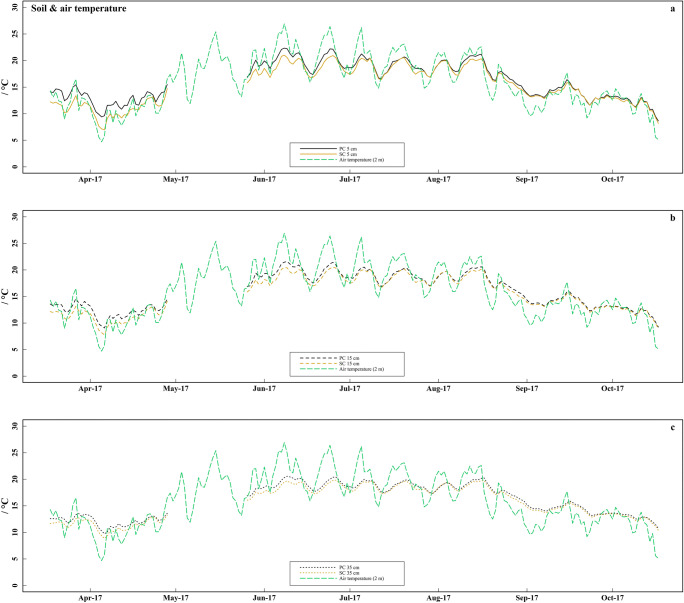
Soil and air temperature. **a**–**c** Daily mean soil temperature in strawberry cultivation, measured at 5, 15, and 35 cm soil depth under plastic coverage (PC) and straw coverage (SC) and daily mean air temperature measured 2 m above ground. The soil temperature data exhibit a data gap from mid-May (13.5) to early-June (8.6), due to technical malfunction of the measuring station

**Fig. 3:**
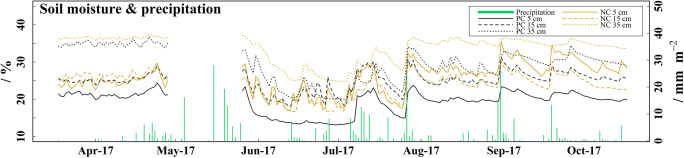
Daily mean soil moisture in strawberry cultivation, measured at 5, 15, and 35 cm soil depth under plastic coverage (PC) and straw coverage (SC) and daily precipitation. The soil moisture data exhibit a data gap from mid-May (13.5) to early-June (8.6), due to technical malfunction of the measuring station

**Fig. 4:**
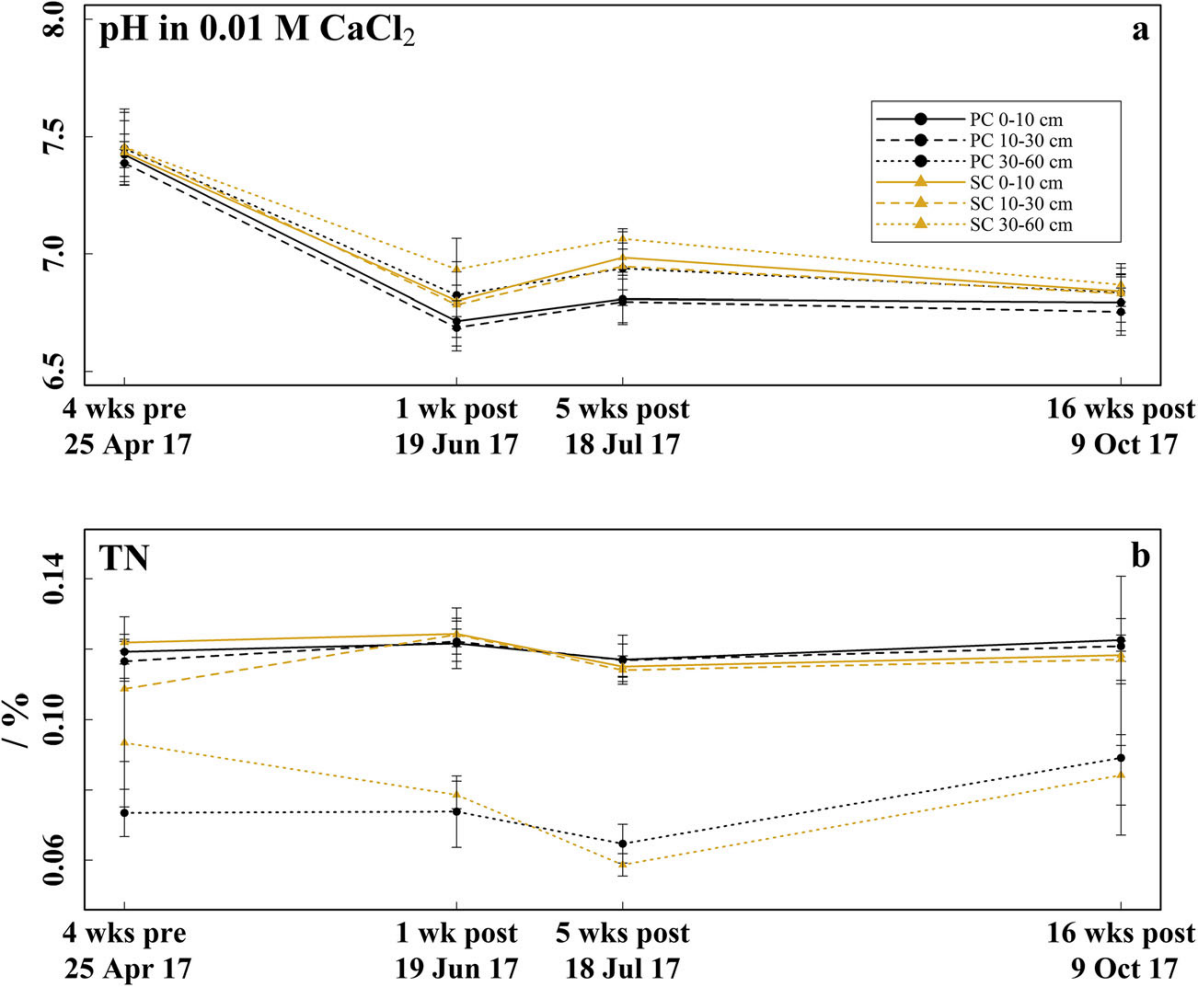
Physicochemical soil properties. **a** pH (in 0.01 M CaCl_2_) determined 4 weeks before (late-April) and respectively 1 week (mid-June), 5 weeks (mid-July), and 16 weeks (mid-October) after fungicide application in the 0–10, 10–30, and 30–60 cm soil layer under plastic coverage (PC) and straw coverage (SC), respectively, shown as mean with standard deviation (*n* = 5). **b** Total nitrogen (TN)

Soil temperature (Fig. 2) was higher under PC than under SC at each soil depth from April to October 2017. The differences in the monthly mean soil temperature (Δ*T*) between both coverage types were up to 2.1 °C at 5 cm soil depth in April and generally decreased with soil depth and during the sampling period (Table 3). Soil moisture (Fig. 3) was markedly lower under PC than under SC at 5 and 35 cm soil depth, whereas at 15 cm soil depth, soil moisture was partially higher under PC. Soil moisture increased with soil depth in both treatments. The pH (Fig. 4a) was between 0.05 and 0.15 units higher under SC compared to PC in all soil layers from mid-July to mid-October. The pH dropped from 7.4 in late-April to values between 6.6 and 7.0 in mid-June in all soil layers of both treatments (*p* < 0.001). The TN (Fig. 4b) ranged between 0.06 and 0.12% but showed no differences between treatments or sampling time.

**Table 3:** Monthly mean, maximum, and minimum soil temperature measured at 5, 15, and 35 cm soil depth by field measuring station under plastic coverage (PC) and straw coverage (SC) during the sampling period. Values are given as mean with standard deviation (values from 13 May to 8 June are missing due to malfunction of the field measuring station)

Date	Soil depth	PC			SC			Δ*T* PC-SC (°C)
		Mean temperature (°C)	Max. temperature (°C)	Min. temperature (°C)	Mean temperature (°C)	Max. temperature (°C)	Min. temperature (°C)	
Apr. 17	5 cm	12.6 ± 2.9	21.7	5.9	10.5 ± 2.2	16.5	4.5	2.1
	15 cm	12.2 ± 1.7	16.7	7.6	10.8 ± 1.6	14.9	6.3	1.4
	35 cm	12.0 ± 1.1	14.3	9.2	11.1 ± 1.0	13.4	8.5	0.9
May 17	5 cm	13.3 ± 1.9	19.3	9.6	12.2 ± 1.6	16.3	8.5	1.2
	15 cm	12.8 ± 1.1	15.8	10.7	12.2 ± 1.1	15.2	9.8	0.6
	35 cm	12.6 ± 0.6	14.3	11.3	12.2 ± 0.7	14.0	11.0	0.4
Jun. 17	5 cm	20.0 ± 1.9	23.9	15.2	18.6 ± 1.8	22.4	14.3	1.4
	15 cm	19.5 ± 1.4	22.4	16.0	18.5 ± 1.4	21.1	15.2	1.0
	35 cm	19.0 ± 1.1	20.7	16.5	18.2 ± 1.1	19.9	15.8	0.8
Jul. 17	5 cm	19.4 ± 1.8	24.7	16.2	18.7 ± 1.5	22.1	15.8	0.7
	15 cm	19.1 ± 1.3	22.4	16.5	18.6 ± 1.2	21.3	16.3	0.5
	35 cm	18.9 ± 0.9	20.7	17.3	18.5 ± 0.9	20.1	17.0	0.4
Aug. 17	5 cm	19.4 ± 1.5	22.8	16.2	19.0 ± 1.4	21.7	15.7	0.4
	15 cm	19.1 ± 1.1	21.3	16.8	18.8 ± 1.1	20.9	16.5	0.3
	35 cm	19.0 ± 0.8	20.5	17.2	18.7 ± 0.7	20.1	17.0	0.3
Sep. 17	5 cm	15.4 ± 1.8	19.9	11.6	15.0 ± 1.7	19.3	11.5	0.4
	15 cm	15.5 ± 1.7	20.3	12.6	15.2 ± 1.6	19.7	12.4	0.3
	35 cm	16.0 ± 1.6	20.3	13.8	15.7 ± 1.6	19.9	13.5	0.3
Oct. 17	5 cm	12.6 ± 1.4	16.8	7.6	12.5 ± 1.4	16.5	7.0	0.1
	15 cm	12.8 ± 1.2	16.2	8.9	12.7 ± 1.2	15.8	8.4	0.1
	35 cm	13.4 ± 1.0	16.0	10.7	13.2 ± 1.0	15.7	10.2	0.2

### Microbial soil parameters

**Fig. 5:**
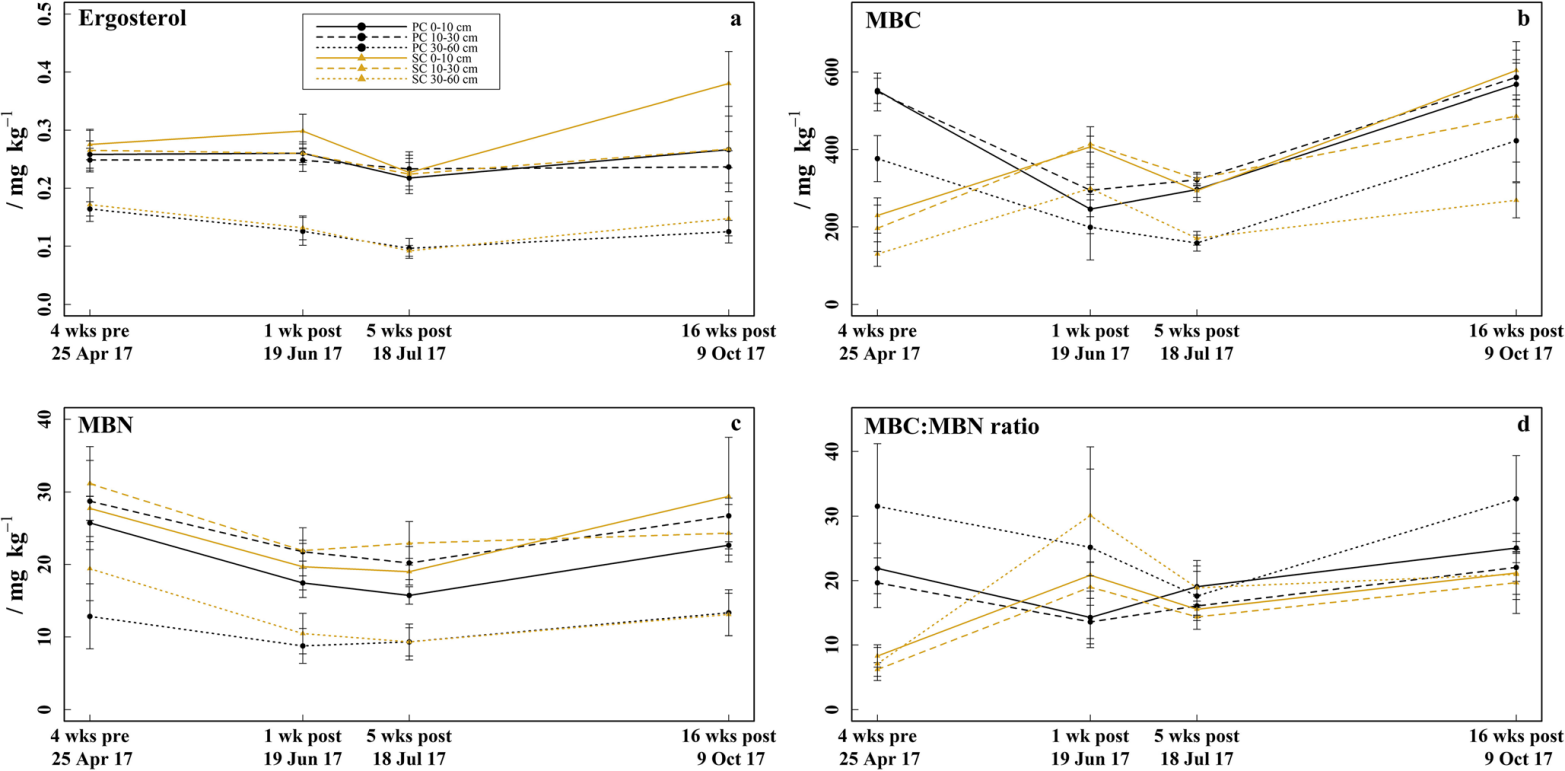
Soil microbial biomass. **a** Ergosterol concentrations determined 4 weeks before (late-April) and respectively 1 week (mid-June), 5 weeks (mid-July), and 16 weeks (mid-October) after fungicide application in the 0–10, 10–30, and 30–60 cm soil layer under plastic coverage (PC) and straw coverage (SC), respectively, shown as mean with standard deviation (*n* = 5). **b** Soil microbial biomass carbon (MBC). **c** Soil microbial biomass nitrogen (MBN). **d** MBC:MBN ratio

The ergosterol concentrations (Fig. 5a) ranged from 0.09 to 0.38 mg kg^−1^ and were by 0.01–0.11 mg kg^−1^ higher under SC compared to PC in the 0–10 cm soil layer (significant in mid-June: *p* = 0.041 and mid-October: *p* = 0.008). The ergosterol concentrations in all soil layers decreased by 0.01–0.04 mg kg^−1^ under PC and by 0.04–0.07 mg kg^−1^ under SC from mid-June to mid-July and increased by 0.01–0.05 mg kg^−1^ under PC and 0.04–0.15 mg kg^−1^ under SC to mid-October. This pattern was most pronounced in the 0–10 cm soil layer under SC. The highest ergosterol concentrations (0.22–0.38 mg kg^−1^) were found in the 0–10 cm soil layer and decreased significantly with soil depth in both treatments (*p* < 0.012).

The MBC (Fig. 5b) ranged from 158 to 604 mg kg^−1^ and was by 247–352 mg kg^−1^ higher under PC than under SC in all soil layers in late-April (*p* < 0.001) and by 100 and 154 mg kg^−1^ higher in the 10–30 and 30–60 cm soil layer in mid-October (30–60 cm soil layer: *p* = 0.001). In contrast, the MBC in mid-June was by 100–161 mg kg^−1^ smaller under PC than under SC in all soil layers (0–10 cm and the 10–30 cm soil layer: *p* ≤ 0.045). Under PC, the MBC decreased in all soil layers from 357 to 552 mg kg^−1^ in late-April to 199–246 mg kg^−1^ in mid-June (*p* = 0.003) and showed less variation to mid-July. From mid-July to mid-October, the MBC under PC increased to 423–586 mg kg^−1^ (*p* < 0.001). In contrast to PC, the MBC under SC increased in all soil layers from 129 to 229 mg kg^−1^ in late-April to 299–414 mg kg^−1^ in mid-June (*p* = 0.001) but showed the same pattern as under PC for mid-July and mid-October.

The MBN (Fig. 5c) ranged from 9 to 31 mg kg^−1^ and was higher under SC than under PC in the 0–10 cm soil layer, with differences increasing from 2.02 to 6.76 mg kg^−1^ during the sampling period. The MBN in all soil layers of both treatments decreased from mid-April to mid-June (*p* < 0.001), remained almost constant until mid-July, and increased from mid-July to mid-October (*p* < 0.001).

The MBC:MBN ratios (Fig. 5d) ranged between 6 and 33 and were significantly wider under PC compared to SC in all soil layers in late-April (*p* ≤ 0.012), whereas in mid-June, the MBC:MBN ratios were by 5–7 units narrower under PC (0–10 and 10–30 cm soil layer: *p* ≤ 0.019). Furthermore, the MBC:MBN ratios were by 2–4 units wider under PC than under SC in the 0–10 and 10–30 cm soil layer in mid-July and by 2–12 units wider under PC in all soil layers in mid-October (30–60 cm soil layer: *p* = 0.039).

**Fig. 6:**
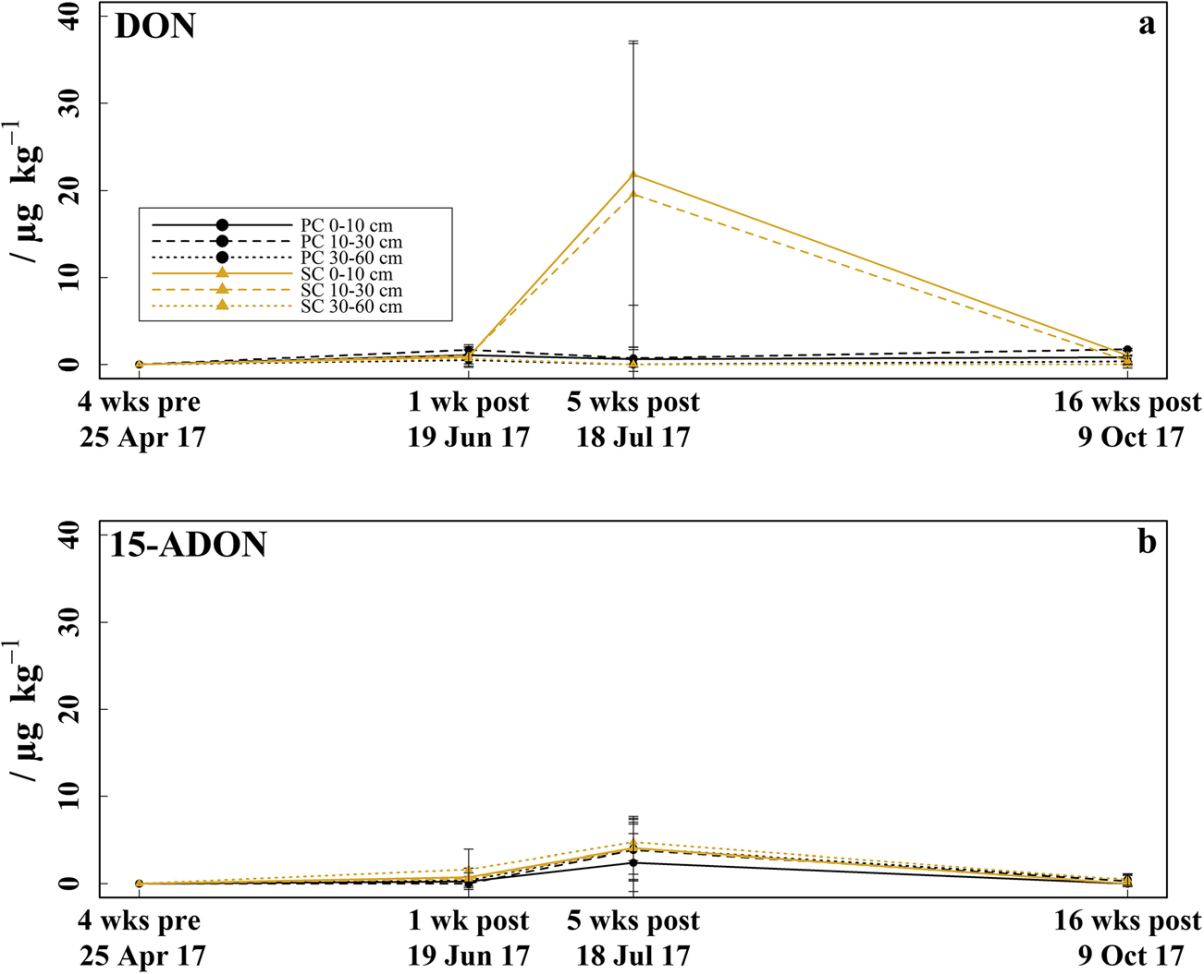
Mycotoxins. **a** Deoxynivalenol concentrations (DON) determined 4 weeks before (late-April) and respectively 1 week (mid-June), 5 weeks (mid-July), and 16 weeks (mid-October) after fungicide application in the 0–10, 10–30, and 30–60 cm soil layer under plastic coverage (PC) and straw coverage (SC), respectively, shown as mean with standard deviation (*n* = 5). **b** 15-Acetyldeoxynivalenol concentrations (15-ADON)

The fusarium mycotoxins DON and 15-ADON (Fig. 6a, b) were measured in concentrations between LCL–21.8 μg kg^−1^ and LCL–4.7 μg kg^−1^ after fungicide application but were not detected in any sample before fungicide application. Both mycotoxins showed a tendency to higher concentrations under SC compared to PC 5 weeks after fungicide application. The DON concentrations in the 0–10 and 10–30 cm soil layer under SC increased in mid-June from slightly above the LCL to ~ 20 μg kg^−1^ in mid-July and then decreased again until mid-October to a level comparable with mid-June. The same pattern was found for 15-ADON in all soil layers of both treatments, but with maximum concentrations ~ 4 μg kg^−1^ in mid-July, the 15-ADON concentrations were markedly lower than the DON values. The mycotoxin NIV was found in none of the soil samples and ZEN was detected only twice in concentrations of 0.6 and 1.1 μg kg^−1^. Cyprodinil and DON showed a positive correlation (*r*_S_ = .380, *p* = 0.029, *n* = 33).

### Soil organic matter

**Fig. 7:**
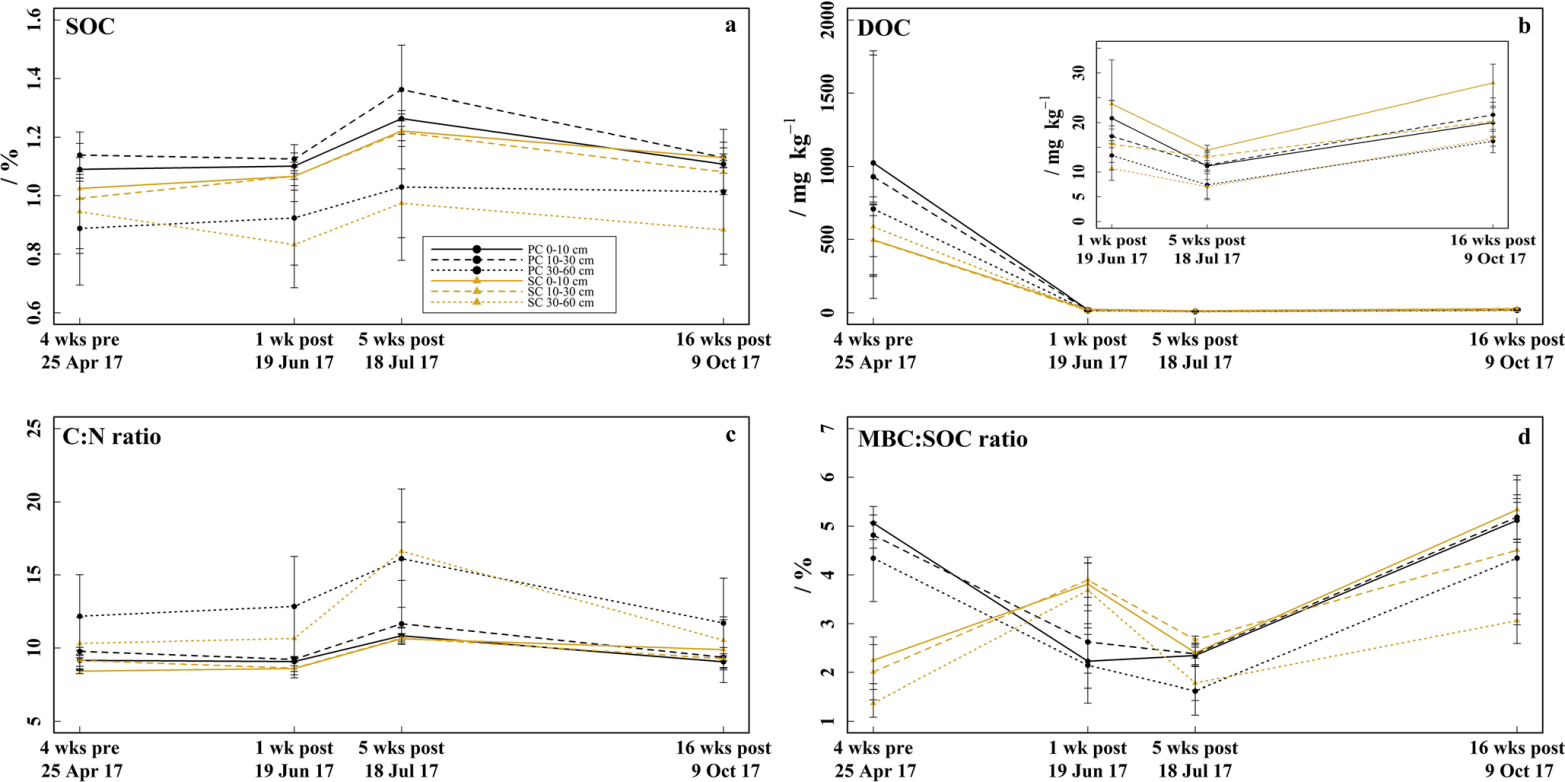
Soil organic matter. **a** Soil organic carbon (SOC) determined 4 weeks before (late-April) and respectively 1 week (mid-June), 5 weeks (mid-July), and 16 weeks (mid-October) after fungicide application in the 0–10, 10–30, and 30–60 cm soil layer under plastic coverage (PC) and straw coverage (SC), respectively, shown as mean with standard deviation (*n* = 5). **b** Dissolved organic carbon (DOC). **c** Carbon:nitrogen ratio (C:N ratio). **d** MBC:SOC ratio

The SOC (Fig. 7a) ranged between 0.83 and 1.36% and was 0.03–0.15% higher under PC in relation to SC in all soil layers during the sampling period (exception: 30–60 cm soil layer in late-April). The SOC increased by 0.11–0.24% in all soil layers of both treatments from mid-June to mid-July (*p* = 0.017) and decreased by 0.02–0.23% in all soil layers of both treatments from mid-July to mid-October (*p* = 0.013).

Because the DOC values (Fig. 7b) in late-April were unrealistically high (494–1024 mg kg^−1^) and we cannot exclude an error during DOC measurement (maybe no filtration), we excluded them from statistical analysis. From mid-June to mid-October, the DOC was 3–8 mg kg^−1^ higher under SC compared to PC in the 0–10 cm soil layer (mid-October: *p* = 0.021). The DOC in all soil layers of both treatments decreased from 11–24 mg kg^−1^ in mid-June to 7–14 mg kg^−1^ in mid-July (*p* < 0.001) and increased again to 16–28 mg kg^−1^ in mid-October (*p* < 0.001).

The C:N ratios (Fig. 7c) increased from mid-June to mid-July (*p* < 0.001) and decreased to mid-October (*p* < 0.001) in all soil layers of both treatments. This was most pronounced in the 30–60 cm soil layer, where C:N ratios increased from 11–12 in mid-June to 16–17 in mid-July and decreased again to 11–12 in mid-October, whereas in the 0–10 cm and the 10–30 cm soil layer, only an increase from 9 to 11–12 and back to 9–10 was observed during the same time period.

The MBC:SOC ratios (Fig. 7d) were 2.81–2.98% higher under PC than under SC in all soil layers in late-April (*p* ≤ 0.003), whereas 1.27–1.58% lower MBC:SOC ratios were observed under PC in mid-June (*p* ≤ 0.030). Under PC, the MBC:SOC ratios were between 4.3 and 5.1% in all soil layers in late-April and decreased to 2.1–2.6% in mid-June (*p* = 0.004), remained almost constant to mid-July, and increased to 4.6–5.2% in mid-October (*p* < 0.001). Under SC, the MBC:SOC ratios were between 1.4 and 2.3% in all soil layers in late-April and increased to 3.7–3.9% in mid-June (*p* = 0.002). Subsequently, the MBC:SOC ratios decreased to 1.8–2.7% in mid-July (*p* < 0.001) and increased to 3.1–5.3% in mid-October (*p* < 0.001).

## Discussion

### Influence of plastic and straw coverage on residual concentration and fate of fungicides in soil

The non-detects of fludioxonil and cyprodinil in late-April confirm that the fungicide concentrations measured after fungicide application were not biased by potential fungicide residues from previous-year applications. The partly two to three times higher fludioxonil and cyprodinil concentrations under SC than under PC in the 0–10 and 10–30 cm soil layer confirmed our hypothesis 1 that the impermeable plastic mulch reduces fungicide entry in soil. However, a significant fraction of the fungicides also reached the soil under PC, presumably through the planting holes. No fenhexamid was detected in any soil sample, which we explain by the fast microbial degradation of fenhexamid under aerobic conditions in soil, resulting in a low DT_50_ of approximately 1 day (Abbate et al. 2007; Borzì et al. 2007).

Although the soil under SC received higher fungicide loads (fludioxonil and cyprodinil), the fungicide concentrations 4 months after fungicide application were similar in both treatments, which point to a faster fungicide degradation under SC, especially in the 0–10 cm soil layer. In average, 27% of the fungicide concentrations measured 1 week after fungicide application were still present in soil 4 months later (without cyprodinil under PC), which point to a slower degradation as reported elsewhere (DT_50_ = 6–21 days for fludioxonil and 2–45 days for cyprodinil) (Agriculture, and Environment Research Unit, University of Hertfordshire 2019; Liu et al. 2011a, b; Zhang et al. 2015). However, the majority of fungicide residues (up to 82%) in soil vanished from mid-June to mid-October, which can be attributed to microbial degradation or non-extractable residue binding to soil matrix and organic matter (Arias et al. 2005; Chen et al. 2018; Dec et al. 1997; Zhang et al. 2015). Because fludioxonil and cyprodinil have both low water solubility and hence a low leachability index (Table 1), leaching processes are unlikely to explain the fungicide declines. According to this, no hints were observed which point to relocation processes of both fungicides with soil depth over time. However, the small fungicide amounts found in the 30–60 cm soil layer 1 week after fungicide application might be explained by preferential flow pathways (Flury 1996; Köhne et al. 2009).

The increase of the cyprodinil concentrations under PC from mid-July to mid-June might be caused by washing off of cyprodinil, which got adsorbed to the plastic film during fungicide application, by the rainfall events between mid-June and mid-July. Many (lipophilic) pesticides have been shown to adsorb to plastic mulches (Guo et al. 2020; Nerín et al. 1996). Thus, an adsorption of fludioxonil and cyprodinil on plastic mulch during spraying seems likely to occur as both are lipophilic (log *K*_ow_ ≥ 4) and exhibit a strong adsorption behavior (Agriculture, and Environment Research Unit, University of Hertfordshire 2019; Arias et al. 2005). Arias et al. (2005) described a higher tendency to desorb again for cyprodinil than for fludioxonil, possibly due to its higher water solubility (Table 1), which might explain why the fungicide increase under PC to mid-July was only found for cyprodinil.

Despite its lower application amount, the cyprodinil:fludioxonil ratios below 1.5 one week after fungicide application indicate an in relation higher fludioxonil concentration compared to cyprodinil, which is in contrast to lower DT_50_ in the field, reported for fludioxonil (6–21 days) than for cyprodinil (2–45 days) (Agriculture, and Environment Research Unit, University of Hertfordshire 2019; Liu et al. 2011a, b; Zhang et al. 2015). A possible reason might be the lower water solubility of fludioxonil (Table 1) and thus its lesser accessibility to microbial degradation (Arias et al. 2005; Roberts et al. 1998). The successive desorption of cyprodinil from the plastic film might be responsible for the increased cyprodinil:fludioxonil ratios under PC 5 weeks and 4 months after fungicide application. Further studies addressing fungicide runoff from plastic-covered ridges and adsorption/desorption processes to plastic mulches are advisable to fully evaluate the influence of plastic mulches on fungicide fate.

Additionally, differences in microclimate, pH, and soil microbial biomass between treatments could have influenced degradation efficiency of fungicides and hence their residual concentrations in soil. Although available literature regarding the degradation of fludioxonil and cyprodinil residues in soil is scarce, it is assumed that soil temperature and moisture correlate positively with fungicide degradation (Fenoll et al. 2010; Liu et al. 2011a; Marín et al. 2003; Roberts et al. 1998). As fungicide residue degradation was mostly faster under SC than under PC, the higher soil temperature under PC obviously not accelerated degradation or this was probably balanced out by the mostly lower soil moisture under PC. Furthermore, soil microbial biomass and activity as well as SOC can influence the degradation of fludioxonil and cyprodinil in soil (Arias et al. 2005; Beigel et al. 1999; Roberts et al. 1998). Thus, the lower MBC under PC in mid-June might decreased fungicide degradation. Additionally, the lower pH under PC than under SC might have induced larger fractions of positive charged fludioxonil and cyprodinil molecules, which adsorb stronger to the soil matrix and to the larger SOC fraction under PC (Arias et al. 2006; Pose-Juan et al. 2011). This can reduce the accessibility of fungicide residues to degradation processes and thus additionally decelerate their degradation under PC.

### Influence of residual concentration of fungicides in soil under plastic and straw coverage on fungi and mycotoxins

Fungal population decreased from mid-June to mid-July as indicated by declining ergosterol concentrations, which was most likely induced by the fungicide residues (Pal et al. 2005; Tu 1993). The effect of fungicide residues on fungi seems to be concentration-dependent as largest declines in fungal populations were found in the topsoil (0–10 cm) under SC, followed by PC, which coincided with the highest loads of fungicide residues. The strong decline in ergosterol from mid-June to mid-July might be explained by the fact that ergosterol is not immediately degraded after fungal cell dead but only after several days (Zhao et al. 2005). However, fungal population recovers within the experimental period as indicated by ergosterol concentrations in mid-October comparable to the initial ones in late-April. The higher ergosterol concentrations under SC than under PC in the 0–10 cm soil layer in mid-June and mid-October might result from the wide C:N ratio of the (wheat) straw mulch, which favor fungal growth (Bossuyt et al. 2001; Muñoz et al. 2017).

Because bacterial biomass have smaller C:N ratios (≈ 4) than fungal biomass (≈ 10) (Sylvia et al. 2005), decreasing MBC:MBN ratios are indicative for decreasing fungal fractions in microbial communities (Campbell et al. 1991). Thus, the smaller MBC:MBN ratios and the larger MBN under SC in the topsoil, 5 weeks and 4 months after fungicide application, point to a shifted microbial community with larger bacterial and smaller fungal fractions. This could be initiated by a stronger intermediate dying-off of fungi under SC due to higher fungicide residues, which were reported elsewhere to favor strongly bacterial proliferation (Martınez-Toledo et al. 1998; Monkiedje 2002) and thus can shift microbial community (Chen et al. 2001a, b; Sigler and Turco 2002). The initially larger MBC under PC compared to SC in late-April might result from higher soil temperatures (> 2 °C) under PC. The lower MBC in mid-June and mid-July compared to mid-October are eventually a combined result from high soil temperature, low soil moisture, and the impact of the fungicide residues on microbial biomass, especially fungal biomass (Lal 2006; Pal et al. 2005; Smith et al. 1993; Yang et al. 2011).

The highest concentrations of the mycotoxins DON and 15-ADON 5 weeks after fungicide application coincide with the lowest values observed for ergosterol. The higher concentrations of DON and 15-ADON observed after fungicide application might be interpreted as a stress response of certain fungi strains to fungicide residues (Jouany 2007; Magan et al. 2002; Ponts 2015). Although literature is scarce on this topic, an increased mycotoxin production of DON and fumonisin B by certain *Fusarium* species after application of fungicides such as myclobutanil, azoxystrobin, and prothioconazole have already been reported for some crops (Audenaert et al. 2011; Li et al. 2018a, b; Simpson et al. 2001). The tendency to higher DON and 15-ADON concentrations under SC may point to a fungicide-induced mycotoxin production, which is corroborated by the significant positive correlation between cyprodinil and DON. But as mycotoxins can also result from stress induction by environmental and biological factors such as high temperatures, water or nutrient scarcity and competition (Medina et al. 2015; Reverberi et al. 2010; Schmidt-Heydt et al. 2008, 2011), maybe high soil temperatures and low soil moisture have additionally triggered mycotoxin formation. However, the investigation if the aforementioned conditions can also influence the occurrence of further mycotoxins such as ochratoxin A, fumonisin Β1, or even aflatoxins, the latter are more relevant in hotter climates (Moretti et al. 2019; Sweeney and Dobson 1998) was beyond the scope of this study but should be investigated in further studies.

In summary, the higher fungicide concentrations under SC stronger reduced fungal biomass in the topsoil (0–10 cm) and induced a higher mycotoxin occurrence of DON and 15-ADON 5 weeks after fungicide application. Thus, a fungicide concentration–dependent effect (and thus coverage type–dependent effect), which was proposed in hypothesis 2, was at least to some extent recognizable, especially in the topsoil where the differences between fungicide residues were largest. However, these effects were short-lived and not observed anymore 4 months after fungicide application.

### Influence of residual concentrations of fungicides in soil under plastic and straw coverage on soil organic matter decomposition

The increase in SOC from mid-June to mid-July might result from a reduced SOM degradation due to an inhibited and reduced microbial biomass by the fungicide residues (Chen et al. 2001a, 2001b; Chen and Edwards 2001). This assumption is corroborated by the in parallel occurring increase in C:N ratio and the decrease of DOC. DOC originates mainly from SOM degradation (Bolan et al. 2011) and can thus be used as an indicator for it. Furthermore, higher C:N ratios can be indicative for an accumulation of fresh biomass, which show normally higher C:N ratios and an easy accessibility for degradation by microorganisms (Blume et al. 2016). Especially fungi play an important role in the degradation of fresh biomass, which favor biomass with large C:N ratios because of their higher C demand (Bossuyt et al. 2001; Keiblinger et al. 2010). In mid-July, the MBC:SOC ratio is partly below 2, which is seen as critical for soil fertility (Anderson 2003) and indicates a low conversion of SOM to MBC and a low SOM degradation (Sparling 1992). However, as confirmed by the strongly increased MBC:SOC ratio in mid-October, the low MBC:SOC ratios might indicate a temporary effect of the fungicide residues on microbial biomass and SOM decomposition.

In summary, our results indicate that the fungicide residues temporarily inhibit and reduce microbial biomass and thus decelerate SOM decomposition, which confirms hypothesis 3. However, no concentration-dependent, and thus treatment-dependent, effect of the fungicide residues could be observed. It can be discussed that the reducing effect on microbial biomass by the fungicide residues might have additionally been enhanced by higher soil temperatures and lower soil moisture during the summer months (Andrade et al. 2003; Smith et al. 1993). However, continuous irrigation by the farmer should have kept soil moisture high enough to maintain microbial growth and activities.

## Conclusion

PC reduces the entry of fludioxonil and cyprodinil into soil after fungicide application compared to SC. The higher fludioxonil and cyprodinil residues under SC strongly reduced the fungal biomass in the topsoil and enhanced the DON and 15-ADON concentrations 5 weeks after fungicide application, which can be interpreted as stress response of the fungal community to the fungicides. Fungal populations were recovered within 4 months, but indications were found that the higher fungicide concentrations under SC had shifted microbial community towards larger bacterial fractions. Furthermore, SOM decomposition was temporarily reduced under both mulching types, presumably by an inhibited and reduced microbial community due to the fungicide residues. In summary, although PC and SC resulted in different amounts of fungicide residues in soil, their effects (partly coverage-dependent) on microbial biomass, fusarium mycotoxin occurrence, and SOM decomposition were short-lived and vanished within 4 months and seem thus not critical for long-term soil fertility and agricultural productivity. However, whether the fungicides can change the structure of the microbial community, especially the fungal community, needs further investigation. Finally, the delayed cyprodinil entry into soil under PC, presumably due to desorption from the plastic film after rainfalls, advises to address the influence of plastic mulching on fungicide adsorption and desorption in further studies.

## Data Availability

The datasets used and/or analyzed during the current study are available from the corresponding author on a reasonable request.

## Abbreviations

SOM: Soil organic matter
DT_50_: Half-life dissipation time
SC: Straw-covered ridge-furrow system with subsurface drip irrigation
PC: Plastic-covered ridge-furrow system with subsurface drip irrigation
TN: Total nitrogen
LC-HRMS: Liquid chromatography–high-resolution mass spectrometry
LCL: Lowest calibration level
RSD: Relative standard deviation
MBC: Microbial biomass carbon
MBN: Microbial biomass nitrogen
HPLC: High-performance liquid chromatography
LOD: Limit of detection
DON: Deoxynivalenol
15-ADON: 15-Acetyldeoxynivalenol
NIV: Nivalenol
ZEN: Zearalenone
SOC: Soil organic carbon
DOC: Dissolved organic carbon
LSD: Least significance distance
